# The Release of Peripheral Immune Inflammatory Cytokines Promote an Inflammatory Cascade in PCOS Patients *via* Altering the Follicular Microenvironment

**DOI:** 10.3389/fimmu.2021.685724

**Published:** 2021-05-17

**Authors:** Yishan Liu, Hao Liu, Zitao Li, Hualin Fan, Xiumin Yan, Xiao Liu, Jianyan Xuan, Du Feng, Xiangcai Wei

**Affiliations:** ^1^ Guangdong Women and Children Hospital, Guangzhou Medical University, Guangzhou, China; ^2^ Guangzhou Municipal and Guangdong Provincial Key Laboratory of Protein Modification and Degradation, State Key Laboratory of Respiratory Disease, School of Basic Medical Sciences, Guangzhou Medical University, Guangzhou, China; ^3^ Department of Cardiology, School of Medicine, South China University of Technology, Guangzhou, China

**Keywords:** polycystic ovary syndrome (PCOS), granulosa cells (GCs), follicular fluid (FF), microenvironment, NF-κB, NLRP3 inflammasomes

## Abstract

**Background:**

Hormones and immune imbalance are critical factors in polycystic ovary syndrome (PCOS). The alternation of immune microenvironment of oocytes may play a significant role in infertility of PCOS patients.

**Objective:**

This study explores the role of follicular fluid microenvironment change in inflammatory pathways activation of granulosa cells (GCs) in PCOS women infertility.

**Methods:**

We enrolled 27 PCOS patients and 30 controls aged 22 to 38 years who underwent IVF and collected their luteinized granulosa cells (LGCs). Meanwhile, a granulosa-like tumor cell line (KGN) as a cell-model assisted this study. Key inflammatory markers in human ovarian GCs and follicular fluid were detected by RT-qPCR, Western blotting, or ELISA. The KGN cells were treated with follicle supernatant mixed with normal medium to simulate the microenvironment of GCs in PCOS patients, and the inflammation indicators were observed. The assembly of NLRP3 inflammasomes was detected by immunofluorescence techniques. Dihydroethidium assay and EdU proliferation assay were used to detect ROS and cell proliferation by flow cytometry.

**Results:**

Compared with normal controls (n = 19), IL-1β (P = 0.0005) and IL-18 (P = 0.021) in the follicular fluid of PCOS patients (n = 20) were significantly increased. The NF-κB pathway was activated, and NLRP3 inflammasome was formatted in ovarian GCs of PCOS patients. We also found that inflammation of KGN cells was activated with LPS irritation or stimulated by follicular fluid from PCOS patients. Finally, we found that intracellular inflammation process damaged mitochondrial structure and function, which induced oxidative stress, affected cellular metabolism, and impaired cell proliferation.

**Conclusion:**

Inflammatory microenvironment alteration in the follicular fluid of PCOS patients leads to activated inflammatory pathway in GCs, serving as a crucial factor that causes adverse symptoms in patients. This study provides a novel mechanism in the inflammatory process of PCOS.

## Introduction

Polycystic ovary syndrome (PCOS) causes essential public health problems, including reproductive, metabolic, and psychological disorders in women. It is one of the most common diseases among women of childbearing age, and the general prevalence in population is about 5-12% (1, 2). The cause of this syndrome remains unclear, but increasing evidence shows that PCOS may be a complex hereditary disease, and vulnerable to environmental impact, including diet and lifestyle changes (2). Moreover, the current diagnostic criteria for PCOS remain incompletely unified, and Rotterdam criteria are still generally accepted (3). The investigation for mechanisms of PCOS progression will be beneficial to diagnosis and therapies for PCOS.

Increasing shreds of evidence have shown that hormones and immune cells play a crucial role in PCOS progression. PCOS also has cross-talks in the role of these immune factors (4). Studies have confirmed that peripheral blood of PCOS patients has elevated C-Reactive Protein (CRP) levels and a significantly increased number of white blood cells. These phenomena suggested that PCOS may be a chronic low-grade inflammatory disease (5). The low-grade chronic inflammation in PCOS patients is mainly attributed to accumulated visceral fat (2, 4), in which adipocytes undergo necrosis after hypoxia and gather many inflammatory cells to produce numerous inflammatory cytokines (6, 7). Therefore, chronic low-grade inflammation plays an irreplaceable role in PCOS progression.

The follicle is the basic functional unit of oocyte generation and development, it induces human germ cell maturation and ovulation (7, 8). GCs control meiosis in mammalian follicles before ovulation (9, 10). The cytokines secreted by GCs were identified as the main component of follicular microenvironment (11). In physiological conditions, adequate inflammatory stress is necessary for normal follicular development and ovulation and contributes to growth and development of oocytes (12, 13). Before ovulation, GCs have a certain inflammatory and immune-like phenotype that produces prostaglandins, inflammatory cytokines, and chemokines, which promote ovulation and fertilization (14). However, in pathological conditions, the development of oocytes may be restricted, and follicles appear early atresia, resulting in ovulatory dysfunction (15). The further development of chronic inflammation induces mitochondrial dysfunction and affects energy supply to oocytes, leading to ovum quality impairment and especially affected ovulation (16). The inflammatory stress in follicular microenvironment may be the underlying mechanism in PCOS progression that we need to investigate further.

Toll-like receptors (TLRs) recognize different pathogen-related molecular patterns (PAMPs) and play an indispensable role in innate immune response (17). They are the first line of defense against pathogen invasion and play a key role in inflammation and regulation, survival, and proliferation in the immune (18). The pro-inflammatory cytokines, including interleukin-1β (IL-1β), IL-18, PAMPs, and lipopolysaccharide (LPS), are activated and released nuclear factor NF-κB complex through IL-1 receptor type 1(IL-1R) and TLR4, respectively. Subsequently, NF-κB is further activated by phosphorylation transported into the nucleus, and promotes the expression of immune response genes (17–19). NF-κB p65 phosphorylation plays a crucial role in NF-κB activation, which seems to be the optimal selection for its activation (20). NLRP3 (encoding protein 3 containing NOD, LRR, and Pyrin domain) is an intracellular sensor that can recognize endogenous damage-associated molecular patterns (DAMPs), and finally form a cytoplasmic complex called NLRP3 inflammasome with ASC and pro-Caspase-1 (21–23), which regulates the maturation and secretion of IL-1β and IL-18 (21–24). The inflammasome formation includes two stages: the first stage is the priming stage, and the second stage is the activation stage (21). Inflammatory cytokines activate NF-κB to participate in inflammation, cause cascade signal amplification (25), and induce NLRP3 inflammasome formation. This signaling pathway regulates cellular physiological processes, including cell cycle arrest, proliferation, cell death, metabolism, stress response, and aging (26).

Given the critical role of IL-1 in regulating human follicular function (15), NLRP3 inflammasome activation in human ovaries is intimately associated with GCs disorder. Investigating the clear inflammatory pathway is obligatory to avoid low-grade inflammation stress in human ovarian GCs. This study aimed to understand whether inflammatory cascade amplification is launched under follicular microenvironment alteration in GCs from PCOS patients and its outcomes. Meanwhile, we also try to systematically elucidate the possible inflammatory pathways and provide novel ideas for PCOS etiology and disease evolution.

## Materials and Methods

### Clinical Patient Data

This study was approved by Institutional Review Committee of Guangdong Women and Children Health Hospital of Guangzhou Medical University. We collected samples according to ethics committee’s approval from Guangdong Women and Children Hospital of Guangzhou Medical University (number: 202001040) and also obtained informed consent of all patients before starting the study. We collected and separated human follicular fluid from oocytes of women receiving IVF. These patients come from Reproductive Center of Guangdong Women and Children Hospital of Guangzhou Medical University. All PCOS patients included in the case group were diagnosed according to revised Rotterdam inclusion/exclusion criteria.

### Follicular Fluid Acquisition and Separation

Under the same GnRH antagonist protocol, ovarian stimulation and oocyte recovery were performed on non-PCOS and PCOS groups. Before aspiration, we measured individual follicles by two-dimensional ultrasound. When the diameter of one follicle was ≥ 18 mm, we provide 10,000 IU of human chorionic gonadotropin (HCG) to induce ovulation. After 36 h, the contents of follicles induced by patient’s stimulation were aspirated through vagina. This process was carried out under general anesthesia. We aspirated the follicles with a 16-gauge single-cavity needle, and completely absorbed and processed each one. After separating oocytes, follicular fluid (FF) is collected in a sterile test tube.

### Extraction and Culture of Human Ovarian LGCs

The collected FF was transferred into 50 mL sterile centrifuge tubes and centrifuged at 1800 rpm for 10 min at room temperature. The above steps were repeated until the supernatant becomes clear, followed by collection. The precipitation is then resuspended in phosphate-buffered saline (PBS) and transferred to a 15 mL centrifuge tube, centrifuged at 3000 rpm for 10 min at room temperature, and repeated once. A new 15 mL centrifuge tube was prepared, and a 5 mL lymphocyte separation solution (Ficoll) (Biosharp, BL 590, China) was added. The precipitation was resuspended with PBS, and a suspension was made with the same volume as Ficoll, spreading the cell suspension evenly on Ficoll, followed by centrifugation by density gradient at room temperature for 30 min. The cells in the middle layer of separation solution were collected and suspended with PBS, then centrifuged at 1800 rpm for 10 min at room temperature. After washing twice with PBS, the cells were resuspended by complete DMEM/F 12 medium (containing DMEM/F 12, 10% fetal bovine serum, and 1% penicillin/streptomycin). After spreading in a 6-well plate, the cells were cultured in a 37°C CO_2_ incubator.

### KGN Cell Line Culture

Human ovarian cancer granulosa cell line KGN cells (Procell CL-0603) (27) were provided by Procell Life Science&Technology Co., Ltd (Wuhan, China). The KGN cell line was cultured in DMEM/F 12 culture (Gibco) (the medium contains 10% FBS (TransSerum^®^ FQ Fetal Bovine Serum) and 1% penicillin/streptomycin. The cells were planted in a 6-well plate and incubated in a 37°C CO_2_ incubator.

### RNA Extraction Technology

The cells were planted in a 6-well plate and mixed with Trizol for 5 min for cell lysis. 0.2 mL chloroform was added to each 1 mL TRIZOL, shaken for 15 s, and place at room temperature for 2 to 3 min. After centrifuging at 12,000 rpm for 15 min at 4°C, the upper aqueous phase was taken to another Ep tube, and 0.5 mL isopropanol was added to mix well, leaving it at room temperature for 10 min. After centrifuging at 12,000 rpm at 4°C for 10 min, we added 1 mL of 75% ethanol to the mix. Before air drying for 5-10 min, we centrifuge the Ep tube at 7500 rpm at 4°C for 5 min. Finally, 40 μL diethylpyrocarbonate water (DEPC) was added to resuspend RNA.

### RT-qPCR Technology

HiScript^®^ II Reverse Transcriptase master mixing system (Vazyme) was used to convert RNA to cDNA. The PCR reaction mixture includes 18 μL qPCR Master Mix premix ChamQ SYBR (Vazyme, Nanjing, China), 2 μL cDNA sample, 0.4 μL forward primer, and 0.4 μL reverse primer. Then running on the machine, the method follows the instructions on the kit. The threshold period value (Ct) was used to determine the expression level of NLRP3, NF-κB, IL-1β, IL-6, and TLR4; then, β-actin was used as a unified parameter to calculate with the equation 2-△△Ct. The sequences of all primers used in this study are listed as followed:

NF-κB (forward: 5’-TGAACCGAAACTCTGGCAGCTG-3’,
reverse: 5’-CATCAGCTTGCGAAAAGGAGCC-3’);
NLRP3 (forward: 5’-GATCTTCGCTGCGATCAACAG-3’,
reverse: 5’-CGTGCATTATCTGAACCCCAC-3’);
IL-1β (forward: 5’-TTACAGTGGCAATGAGGATGAC-3’,
reverse: 5’-GTGGTGGTCGGAGATTCGTA-3’);
IL-6 (forward: 5’-AGACAGCCACTCACCTCTTCAG-3’,
reverse: 5’-TTCTGCCAGTGCCTCTTTGCTG-3’);
TLR4 (forward: 5’-CCCTGAGGCATTTAGGCAGCTA-3’,
reverse: 5’-AGGTAGAGAGGTGGCTTAGGCT-3’);
β-actin (forward: 5’-GTTGTCGACGACGAGCG-3’,
reverse: ‘5’-GCACAGAGCCTCGCCTT-3’).

### Western-Blotting Technology

The protein extraction process was processed on ice. The cell samples in the 6-well plate were gently washed three times with PBS, and RIPA buffer (Beyotime, Shanghai, China) mixed with 1mM protease inhibitor phenylmethanesulfonyl fluoride (PMSF) (Beyotime, Shanghai, China) and 100X Roche protease inhibitor (cocktail) (Beyotime, Shanghai, China) was added to each dish for 30 min. The cells were transferred to a centrifuge tube, centrifuged at 15000 rpm for 15 min, and the supernatant was saved. BCA kit (Beyotime, China) was used to determine protein concentration). Every 1 μL 6X loading buffer was added in 5 μL protein and heated in a 100°C water bath for 10 min. The gel electrophoresis was performed using a 15% strength performed gel to separate proteins and then transferred to PVDF membrane. A pre-prepared blocking solution (5% BSA) was used to seal the membrane for 1 h, followed by placing them in an anti- 4°C refrigerator overnight. Washing with Tris-buffered saline Tween (TBST) was done three times on the next day, 10 min each time. After washing, a secondary antibody was added and incubated at room temperature for 1 h. The PVDF membrane was successfully contacted with an enhanced chemiluminescence reagent (Biosharp) and exposed to an enhanced chemiluminescence detection system (Amersham, Piscataway, NJ, China). Image J (National Institutes of Health, Bethesda, Maryland, USA) software processing system was employed for optical density analysis.

### Immunofluorescence Staining

The cells were seeded in a 12-well plate in advance, and a cell sheet was spread in each well. When the cell density is 80% - 90%, the culture medium was discarded, the cells were fixed with 4% paraformaldehyde for 25 min, and washed three times with PBS. Then, infiltration was done using 0.5% TritonX-100 (Beyotime, Shanghai, China) for 10 min. After blocking with fluorescent staining blocking solution (Beyotime, Shanghai, China) for 15 min, the cells were washed with PBS three times. The specific antibody was incubated with primary antibody diluent (1:200 Beyotime, Shanghai, China) overnight in an anti- 4°C refrigerator. After washing with PBS on next day, a secondary antibody (1:200) was added to the cells at room temperature for 1 h (in a dark box to avoid light). Then, the cells were washed with PBS and were covered with a mounting medium with DAPI. The cells were observed under a confocal microscope after the mounting tablets were dried (Zeiss LSM 800).

### Enzyme-Linked Immunosorbent Assay (ELISA)

ELISA analysis detects IL-1β and IL-18 levels in the supernatant of isolated follicles. The test was carried out by manufacturer’s agreement by ELISA kit (Solarbio, Beijing, China), and the test was repeated three times for one sample.

### Measurement of ROS by Flow Cytometry Analysis (FCM)

The sample preparation was carried out according to manufacturer’s instructions. The cells were digested with 25% pancreatin and were collected in a 15 mL centrifuge tube. After centrifugation, the supernatants were removed, and cell suspensions were prepared by adding PBS 1 mL of cells suspension mixed with 1μL of DHE or mixed with 2 μL of EdU. The assay reagent and samples were incubated for 30 min (DHE) or 2 h (EdU) at room temperature in dark. All fluorescence signals of labeled cells were analyzed by the flow cytometer CytoFLEX S (Beckman Coulter). A minimum of 10,000 cells were examined for each assay at a 100-150 cells/second flow rate. PI red fluorescence (590–610 nm) or FITC green fluorescence (488-519 nm) was analyzed in FL-2 channel. The percentage of PI-positive or FITC-positive cells were analyzed using flow cytometer software CytExpert, version 2.3.0.84 (Beckman Couter, Inc).

### 5-Ethynyl-2’- Deoxyuridine (EdU) Method to Detect Cell Proliferation

We use BeyoClick™ EdU Cell Proliferation Kit with Alexa Fluor 488 to assess cell proliferation. The test was carried out following the manufacturer’s agreement by this kit (Beyotime, Shanghai, China). After staining a part of cells, the number of stained cells was counted under a confocal microscope and was compared with the count of all cells in the field of view to calculate the proportion of proliferating cells. Three fields of view were counted for each group. The fluorescence intensity of the other part of cells was observed under the flow cytometer to further judge cell enhancement, and the method is explained above. This test was repeated three times for one sample.

### Data Analysis

Statistical analysis was performed using IBM SPSS Statistics 25.0 (SPSS Inc, Chicago, IL, USA), and we used Graphpad Prism 8.3.0 software (Graphpad Software Inc., San Diego, CA, USA) to generate graphs. Qualitative data were expressed as means ± standard error of the mean (SEM) and P-values were analyzed by two independent sample t-tests. It was considered that p < 0.05 is significantly different.

## Results

### Clinical Cases

Before IVF procedure, the patients should perform a general clinical examination. Indicators, including anti-Müllerian hormone (AMH; ng/mL), follicle-stimulating hormone (FSH; IU/L), luteinizing hormone (LH; IU/L), estradiol (E2; ng/mL), progesterone (P; ng/mL), and testosterone (T; ng/mL) were calculated. Besides, the ratio of LH to FSH was also calculated to make a diagnosis. The study results showed no significant difference in age and FSH, E2, and P levels between PCOS infertile patients and controls (**Table 1**, P > 0.05). However, hormone levels such as AMH, T, LH, and LH/FSH ratio in PCOS serum increased significantly compared with controls (P < 0.01). Fasting blood glucose, fasting insulin, triglycerides, cholesterol, and low-density lipoprotein (LDL) were higher than controls and were statistically significant (P < 0.05). We also found that peripheral white blood cell content of PCOS patients was higher than that of normal control group (P = 0.013).

**Table 1:** The clinical characteristics of PCOS patients and controls.

Characteristic	Controls (N=30)	PCOS(N=27)	P value
Age (y)	29.530 ± 3.441	27.190 ± 3.317	0.836
BMI(kg/m^2^)	19.358 ± 0.897	24.561 ± 2.790	<0.0001****
AMH (ng/mL)	3.924 ± 1.984	13.467 ± 3.885	0.003**
Basal FSH (IU/L)	6.757 ± 1.707	5.624 ± 1.638	0.872
Basal LH (IU/L)	5.092 ± 1.470	10.747 ± 5.306	<0.0001****
Basal LH/FSH	0.776 ± 0.240	1.980 ± 0.930	<0.0001****
Basal E2 (ng/L)	45.548 ± 15.901	46.589 ± 12.142	0.238
Basal P (ng/L)	0.231 ± 0.130	0.283 ± 0.133	0.960
Basal T(ng/mL)	0.205 ± 0.077	0.622 ± 0.351	<0.0001***
WBC(g/L)	5.862 ± 0.872	7.348 ± 0.613	0.020*
Fasting blood glucose(mmol/L)	4.957 ± 0.257	5.061 ± 0.359	0.004**
Fasting insulin(uU/m)	7.537 ± 2.123	15.367 ± 10.400	0.0003***
Cholesterol(mmol/L)	4.716 ± 0.673	5.258 ± 0.9655	0.022*
Triglycerides(mmol/L)	0.9601 ± 0.409	1.845 ± 0.972	0.001**
LDL(mmol/L)	2.826 ± 0.516	3.195 ± 0.718	0.011*
HDL(mmol/L)	1.614 ± 0.329	1.419 ± 0.307	0.591

P-values reported are the results of independent sample t-tests for dichotomous variables; x ± s; *P < 0.05, **P < 0.01, ***P < 0.001, ****P < 0.0001. AMH, anti-Müllerian hormone; FSH, follicle-stimulating hormone; LH, luteinizing hormone; E2, estradiol; P, progesterone; T, testosterone; LDL, low-density lipoprotein; HDL, high density lipoprotein.

### The Levels of IL-1β and IL-18 Increased in the Follicular Fluid of PCOS Patients

**Figure 1:**
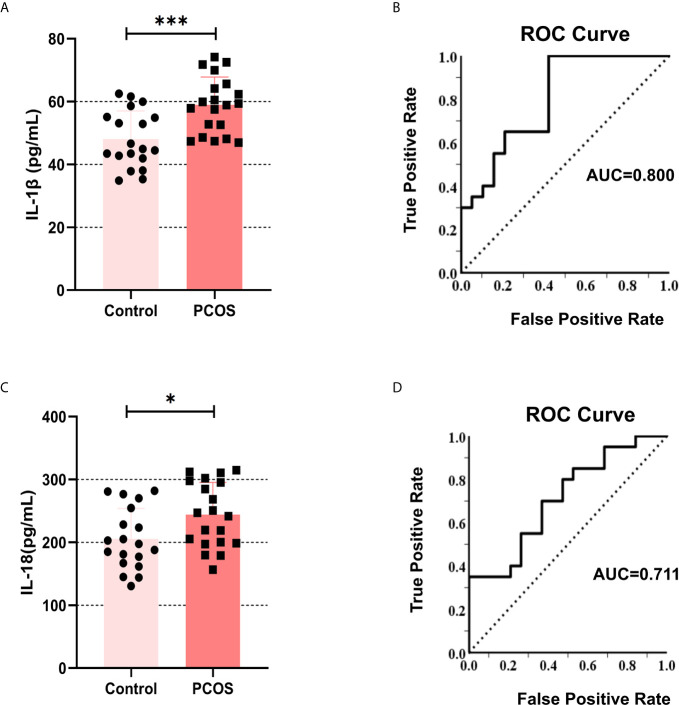
The levels of IL-1β and IL-18 increased in the follicular fluid of the PCOS patients. **(A)** The content of IL-1β in follicular fluid in PCOS patients and controls were measured by ELISA (P = 0.0005). **(B)** ROC analysis for the regression between levels of IL-1β and PCOS diagnosis, AUC = 0.800. **(C)** The content of IL-18 in follicular fluid in PCOS patients and controls were measured by ELISA (P = 0.021). **(D)** ROC analysis for the regression between levels of IL-18 and PCOS diagnosis, AUC = 0.711. *P < 0.05 and ***P < 0.001. *P < 0.05 was considered statistically significant.

To clarify that inflammatory cytokines in the peripheral circulation may accumulate in the follicular fluid through ovarian microcirculation. We collected the follicular fluid of patients undergoing IVF. The primary GCs were isolated for culture, and the supernatant was retained for ELISA detection of IL-1β and IL-18. The results showed that the content of IL-1β (**Figure 1A**) and IL-18 (**Figure 1C**) in the follicular supernatant was higher in PCOS patients compared with controls (IL-1β: P = 0.0005, IL-18: P = 0.021). Simultaneously, we used ROC analysis for the regression between levels of IL-1β and PCOS diagnosis, and we obtained AUC (Area Under Curve) of 0.800 (**Figure 1B**). IL-18 was analyzed in the same way, and we obtained AUC of 0.711 (**Figure 1D**). These data indicate that the content of IL-1β and IL-18 in the follicular fluid of PCOS patients is abnormally increased, which may underpin the pathogenesis of this disorder.

### NF-κB Pathway Activation and NLRP3 Inflammasome Formation Were Promoted in Ovarian GCs From PCOS Patients

**Figure 2:**
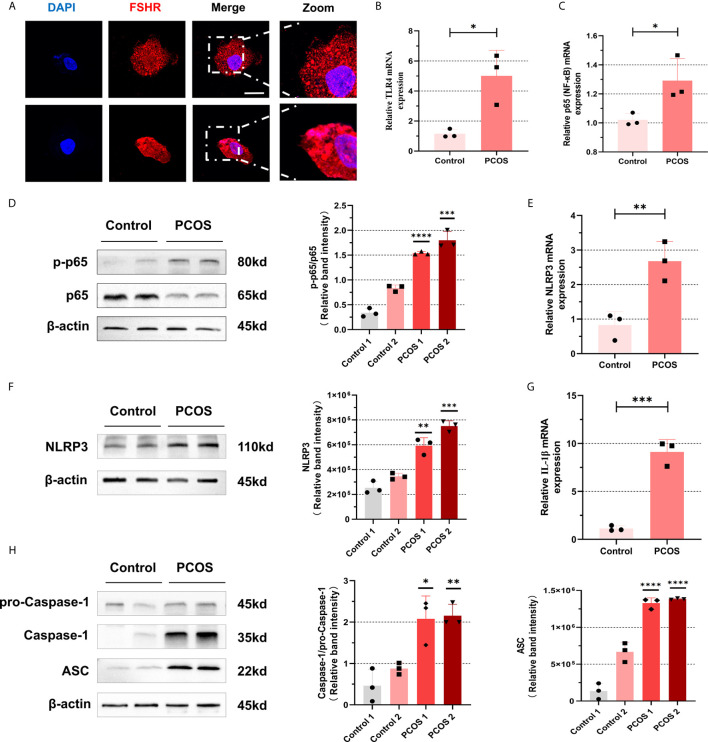
NF-κB pathway activation and NLRP3 inflammasomes formation were promoted in ovarian granulosa cells from PCOS patients. **(A)** Identification of GCs in primary cells extracted from ovaries of PCOS patients and controls by immunofluorescence assays. (FSHR, red; DAPI, blue; scale bar, 20 μm). **(B)** The mRNA levels of TLR4 between PCOS patients and controls in GCs were measured by RT-qPCR assays (P = 0.0184). **(C)** The mRNA levels of p65 between PCOS patients and controls in GCs were measured by RT-qPCR assays (P = 0.0292). **(D)** The phosphorylation levels of p65 were measured by western blotting assays. **(E)** The mRNA levels of NLRP3 in GCs in PCOS patients and controls were measured by RT-qPCR assays (P = 0.0096). **(F)** NLRP3 protein levels in GCs in PCOS patients and controls by western blotting assays. **(G)** The mRNA levels of IL-1β from PCOS patients and controls by RT-qPCR assays (P = 0.0005). **(H)** The levels of NLRP3 inflammasome-related proteins (ASC, pro-caspase-1, and caspase-1) were measured by western blotting assays. *P < 0.05, **P < 0.01, ***P < 0.001 and ****P < 0.0001. *P < 0.05 was considered statistically significant.

To further identify whether follicular microenvironment affects the inflammatory conditions of GCs, we experimented with primary cells extracted from ovaries of PCOS patients and controls. Follicle-stimulating hormone receptor (FSHR) immunocytochemical staining is the specific staining of ovarian GCs (27). We used this method to ensure that we isolated GCs correctly. The FSHR (red) positive staining is localized in cell membrane, suggesting that the method is successful (**Figure 2A**). After confirming that GCs can be successfully separated, we conducted a series of experiments on them. As shown in **Figures 2B, C**, mRNA levels of TLR4 and p65 in GCs of PCOS patients were increased compared with controls (TLR4: P = 0.0184, p65: P = 0.0292). We further confirmed that increased phosphorylation of p65 protein was significant (**Figure 2D**), suggesting that NF-κB inflammatory pathway was activated through TLR4. In addition, we also found that mRNA level of IL-6 in CGs of PCOS patients were increased (P=0.0071) (**Figure S1A**). In PCOS patients, mRNA levels of NLRP3 (P = 0.0096) are higher than that in normal individuals (**Figure 2E**), and the amount of NLRP3 increased equally (**Figure 2F**). To further verify that NLRP3 inflammatory pathway was activated, we found that mRNA expression of IL-1β in PCOS patients was significantly increased (P = 0.0005) (**Figure 2G**). We used WB to detect levels of NLRP3 inflammasome-related protein ASC, pro-Caspase-1, and Caspase-1, and the increase of self-cleavage into a mature form of Caspase-1 was observed (**Figure 2H**).

### NF-κB and NLRP3 Inflammasome of Human Ovarian Granulosa Cells Were Activated After LPS Stimulation

**Figure 3:**
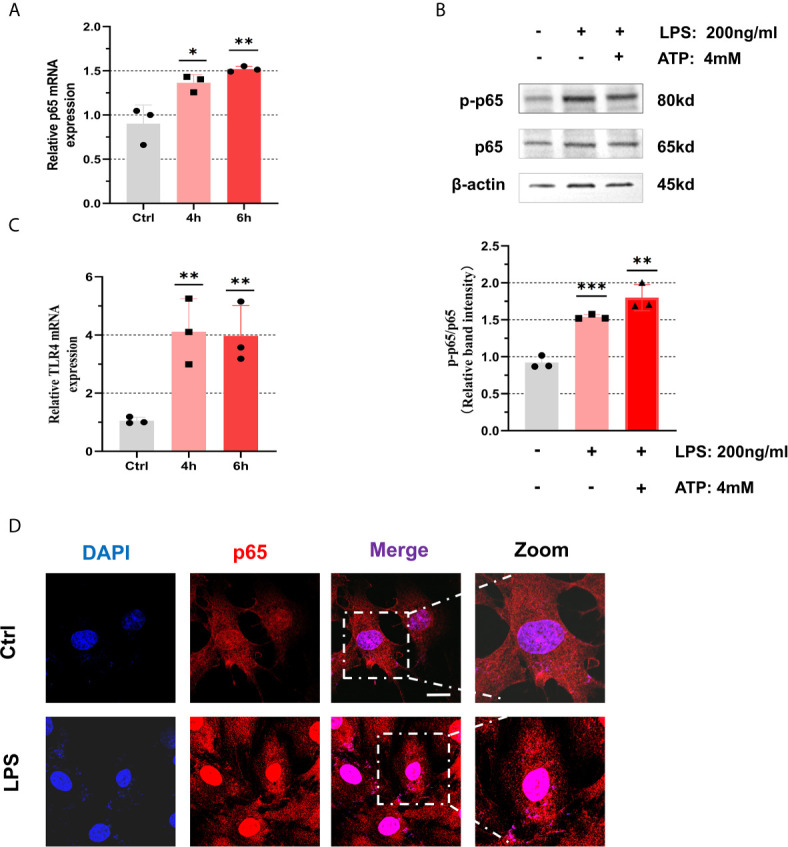
NF-κB pathway was activated with treatment of LPS in the KGN cells. **(A)** After treatment with LPS (200 ng/mL) for 4 h or 6 h, the relative expression of p65 was measured by RT-qPCR (4 h: P = 0.0257, 6 h: P = 0.0074). **(B)** The phosphorylation levels of p65 in KGN cells with the treatment of LPS (200 ng/mL) for 6 h and ATP (4 mM) for 50 min. **(C)** After treatment with LPS (200 ng/mL) for 4 h or 6 h, the relative expression of TLR4 was measured by RT-qPCR (4 h: P = 0.0096, 6 h: P = 0.0086). **(D)** The localization of p65 in KGN cells with LPS (200 ng/mL) stimulation for 3 h by immunofluorescent assays (p65, red; DAPI, blue; scale bar, 20 μm). *P < 0.05, **P < 0.01 and ***P < 0.001. *P < 0.05 was considered statistically significant.

To clarify that GCs can indeed activate NF**-**κB signaling pathway and promote the activation of NLRP3 inflammasomes under positive stimulation, we used LPS (200 ng/mL) and ATP (4 mM) as activators to induce inflammation in KGN cells (28). The results suggested that after incubation with LPS (200 ng/mL) for 4 h and 6 h, mRNA levels of p65 were significantly up-regulated in KGN cells (4 h: P = 0.0257, 6 h: P = 0.0074) (**Figure 3A**). The phosphorylation of p65 protein increased after treatment with LPS (200 ng/mL) for 6 h and ATP (4 mM) for 50 min (**Figure 3B**). We found that mRNA levels of TLR4 in KGN cells after treatment with LPS (200 ng/mL) for 4 h and 6 h were up-regulated (4 h: P = 0.0096, 6 h: P = 0.0086) (**Figure 3C**). We also observed that NF-κB was in an inactive state in cytoplasm at first and then entered into nucleus after being activated (**Figure 3D**). These data show that NF-κB pathway is activated in KGN cells stimulated by LPS. At the same time, we also found that mRNA level of IL-6 increased after LPS treatment (4 h: P = 0.0073, 6 h: P = 0.0374) (**Figure S1B**).

**Figure 4:**
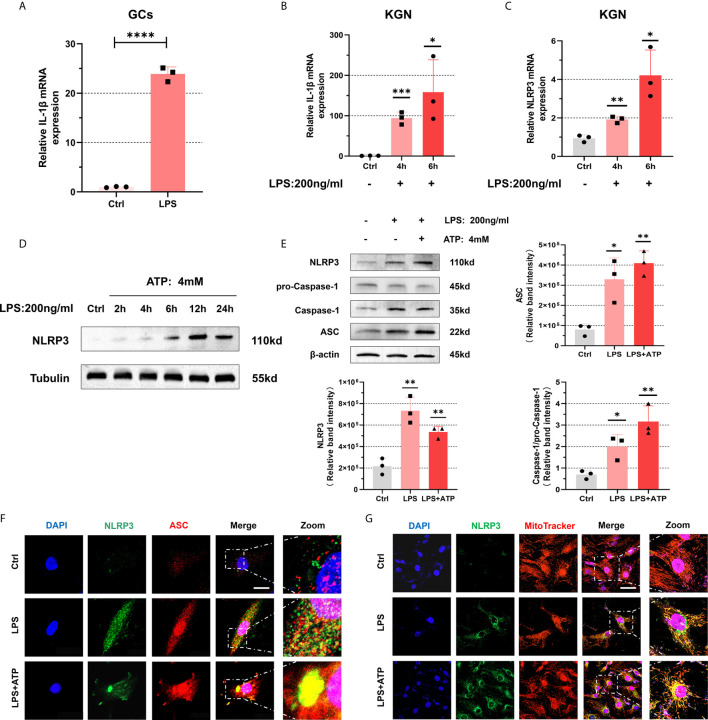
NLRP3 inflammasomes were activated in KGN cells with LPS stimulation. **(A)** The mRNA level of IL-1β in primary human GCs treated with LPS (200 ng/mL) for 4 h was measured by RT-qPCR assays (P < 0.0001). **(B)** With LPS stimulation (200 ng/mL) in KGN cells, IL-1β mRNA levels were detected by RT-qPCR assays (4 h: P = 0.0004, 6 h: P = 0.0271). **(C)** The mRNA level of NLRP3 in KGN cells stimulated with LPS (200 ng/mL) (4 h: P = 0.0024, 6 h: P = 0.0131). **(D)** NLRP3 protein levels in KGN cells with LPS treatment (200 ng/mL) for 2, 4, 6, 12, and 24 h. **(E)** The protein levels of NLRP3, ASC, pro-Caspase-1, and Caspase-1 in KGN cells were treated with LPS (200 ng/mL) for 12 h and ATP (4 mM) for 50 min. **(F)** Immunofluorescent staining for co-localization of NLRP3 with ASC in KGN cells with LPS treatment (200 ng/mL) for 3 h and ATP (4 mM) for 50 min. (NLRP3, green; ASC, red; DAPI, blue; scale bar, 20μm). **(G)** Immunofluorescent staining for co-localization of NLRP3 with mitochondria in the KGN cells with LPS stimulation (200 ng/mL) for 3 h and ATP (4 mM) for 50 min (NLRP3, green; MitoTracker indicated mitochondria, red; DAPI, blue; scale bar, 100μm). *P < 0.05, **P < 0.01, ***P < 0.001 and ****P < 0.0001. *P < 0.05 was considered statistically significant.

Next, we conducted experiments to explore the activation of NLRP3 inflammasomes. After treating with LPS (200 ng/mL) for 4 h, the levels of IL-1β in primary GCs were significantly increased (P < 0.0001) (**Figure 4A**). Similarly, when we use LPS(200 ng/mL) to stimulate KGN cells, an increasing trend emerged in the expression of IL-1β (4 h: P = 0.0004, 6 h: P = 0.0271) (**Figure 4B**). Equally, we detect mRNA level of NLRP3 after its treatment with LPS (200 ng/mL) and found its up-regulation (4 h: P = 0.0024, 6 h: P = 0.0131) (**Figure 4C**). Further evidence indicated that expression of NLRP3 protein increased with prolonged stimulation time and had a remarkable trend with 12 h treatment (**Figure 4D**). So we stimulated the cells with LPS (200 ng/mL) for 12 h to induce inflammation. The expressions of NLRP3, ASC, pro-Caspase-1, and Caspase-1 were up-regulated in KGN cells treated with LPS (200 ng/mL) for 12 h and ATP (4 mM) for 50 min (**Figure 4E**). To investigate whether inflammasomes have been assembled, we observed the co-localization of NLRP3 and ASC in KGN cells under a confocal microscope. We found that with LPS treatment (200 ng/mL) for 3 h and ATP (4 mM) for 50 min, co-localization was observed (**Figure 4F**). Interestingly, we also observed that NLRP3 protein was located on mitochondria and accumulated around the nucleus, which further confirmed the formation of inflammasomes (29) (**Figure 4G**).

### The NF-κB Pathway and NLRP3 Inflammasomes Were Activated in KGN Cells Stimulated With Follicular Fluid From PCOS Patients

**Figure 5:**
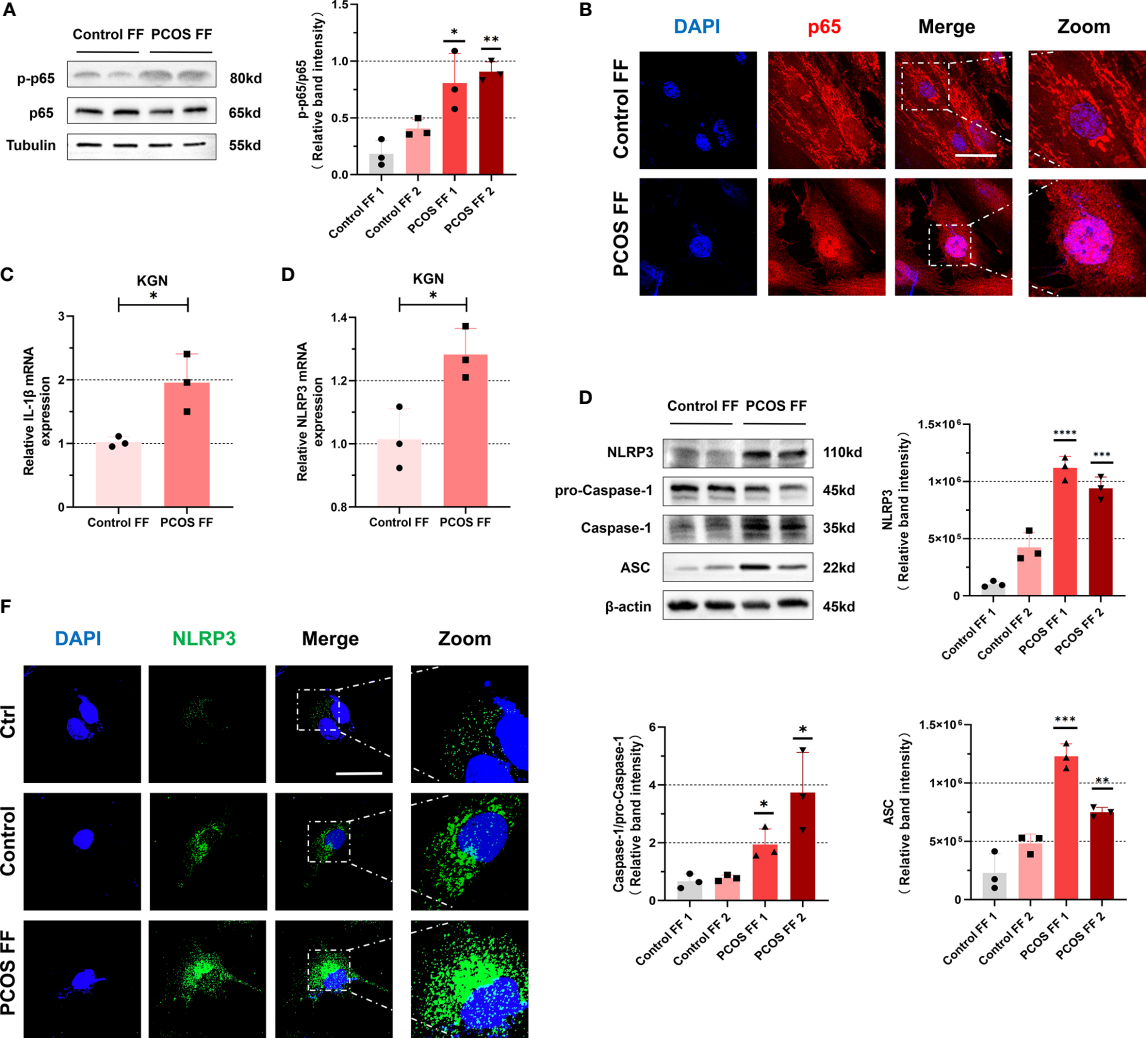
NF-κB pathway and NLRP3 inflammasomes in KGN cells were activated by stimulation of with follicular fluid from PCOS patients. **(A)** With treatment of follicular fluid in KGN cells of PCOS patients and controls for 3 h, the phosphorylation levels of p65 were measured by western blotting assays. **(B)** The localization of p65 in KGN cells with follicular fluid treatment of PCOS patients and controls for 3 h by immunofluorescent assays. (p65, red; DAPI, blue; scale bar, 50 μm). **(C)** The KGN cells were treated with follicular fluid of PCOS patients and controls for 3 h, and IL-1β mRNA levels were measured by RT-qPCR (P = 0.0097). **(D)** The mRNA level of NLRP3 was detected in KGN cells with treatment of follicular fluid of PCOS patients and controls for 3 h (P = 0.0011). **(E)** NLRP3 inflammasome-related proteins (NLRP3, ASC, pro-Caspase-1, and Caspase-1) were measured by western blotting assays. **(F)** With treatment of follicular fluid of PCOS patients and controls for 3 h, the localization of NLRP3 in KGN cells is measured by immunofluorescent assays (NLRP3, green; DAPI, blue; scale bar, 50 μm). *P < 0.05, **P < 0.01, ***P < 0.001 and ****P < 0.0001. *P < 0.05 was considered statistically significant.

To further explore that changes in follicular fluid microenvironment may promote an inflammatory cascade in GCs from PCOS patients, we mix the follicular fluid isolated from patients with a culture medium in a certain ratio of 1:2 (FF: DMEM/F 12) to cultivate KGN cells. We observed that the level of phosphorylated p65 protein in KGN cells increased after 6 h of culture with follicular fluid of PCOS patients (**Figure 5A**). To further clarify our conjecture, we performed NF-κB staining. It revealed that NF-κB protein entered the nucleus and was located in the nucleus after incubating the follicular fluid of PCOS patients (**Figure 5B**). All suggest that NF-κB pathway was activated. To further explore whether NLRP3 inflammasomes were activated, we cultured KGN cells in the same way on the above. After culturing KGN cells with patient’s follicular fluid for 3 h, mRNA level in IL-1β (P = 0.0097) (**Figure 5C**) and NLRP3 (P = 0.0011) (**Figure 5D**) was increased. Besides, NLRP3 inflammasome-related proteins such as NLRP3, ASC, pro-Caspase-1, and Caspase-1 were also significantly increased (**Figure 5E**). Furthermore, under the immunofluorescence confocal microscope, the expression of NLRP3 protein increased (**Figure 5F**). Overall, these data show that activated NF-κB pathway and NLRP3 inflammasomes in KGN cells were stimulated with follicular fluid from PCOS patients.

### Follicular Fluid From PCOS Patients and LPS Impaired Mitochondria Structure and Function, Caused Oxidative Stress, and Arrested Cellular Proliferation

**Figure 6:**
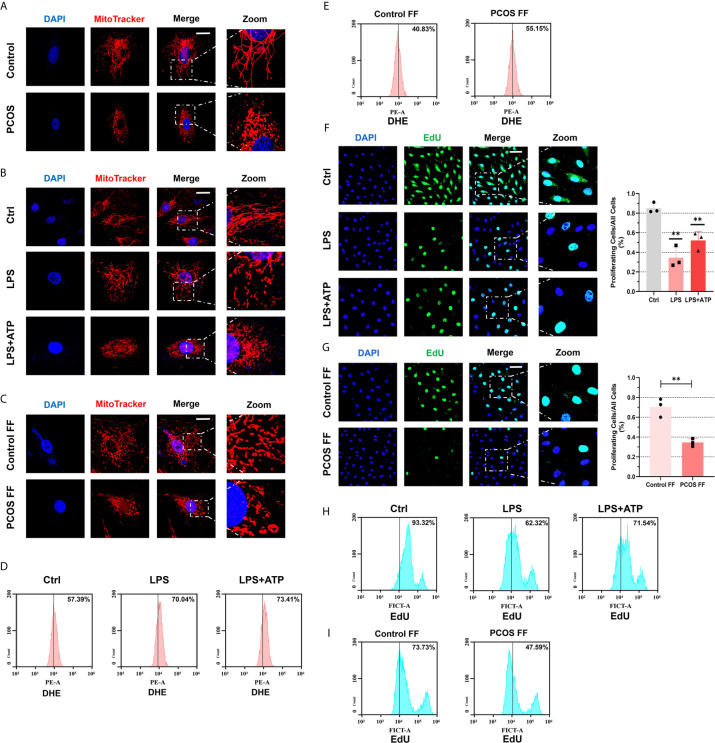
Follicular fluid from PCOS patients and LPS impaired mitochondria structure and function, caused oxidative stress, and arrested cellular proliferation. **(A)** Immunofluorescence imaging of mitochondria in GCs from PCOS patients and controls (MitoTracker, red; DAPI, blue; scale bar, 20 μm). **(B)** Immunofluorescence imaging of mitochondrial morphology in KGN cells with LPS stimulation (200 ng/mL) for 3 h and ATP (4 mM) for 50 min (MitoTracker indicated mitochondria, red; DAPI, blue; scale bar, 20 μm). **(C)** Immunofluorescence imaging of mitochondrial morphology in KGN cells incubated with follicular fluid for 3 h (MitoTracker indicated mitochondria, red; DAPI, blue; scale bar, 20 μm). **(D)** After DHE staining, flow cytometry assays were used to detect ROS levels in KGN cells with LPS (200 ng/mL) treatment for 3 h and ATP (4 mM) for 50 min. **(E)** After DHE staining, flow cytometry assays were used to detect ROS levels in KGN cells with follicular fluid stimulation for 3 h. **(F)** Immunofluorescence imaging of EdU to indicate the KGN cells proliferation with LPS (200 ng/mL) treatment for 3 h and ATP (4 mM) for 50 min (EdU, green; DAPI, blue; scale bar, 50 μm). **(G)** Immunofluorescence imaging of EdU to indicate the KGN cells proliferation with follicular fluid treatment for 3 h (EdU, green; DAPI, blue; scale bar, 50 μm). **(H)** With EdU staining, flow cytometry assays were used to detect fluorescence in KGN cells with LPS (200 ng/mL) stimulation for 3 h and ATP (4 mM) for 50 min treatment. **(I)** After EdU staining, flow cytometry assays were used to detect fluorescence in KGN cells with treatment of follicular fluid for 3 h. **P < 0.01. *P < 0.05 was considered statistically significant.

To further judge the influence of inflammation on cells, we observe the mitochondrial morphology under a confocal microscope. We found that mitochondrial morphology of GCs was fragmented in PCOS patients (**Figure 6A**). Then we stimulated KGN cells with LPS or follicular fluid respectively and observed fragmented mitochondria (**Figures 6B, C**). An article pointed out that fragmented mitochondria produce more ROS (30), so our next experiment tested ROS levels in KGN cells with LPS or follicular fluid treatments. After using DHE to detect ROS and measure it by FCM, we observed an increase in generating ROS after LPS treatment, and the stimulate was more obvious after ATP addition (**Figure 6D**). Follicular fluid in controls seems to have a protective mechanism to inhibit ROS generation, so ROS levels were decreased. Conversely, the anti-oxidation ability of follicular fluid of PCOS patients on the cells is weakened, so ROS decrease is not obvious or even increased (**Figure 6E**). To investigate whether cell growth is impaired, we used EdU proliferation assay to measure cell proliferation. We observed that cell proliferation rate was slowed down after LPS treatment under immunofluorescent assays (**Figure 6F**). The same phenomenon was observed in KGN cells co-cultured with a follicular fluid of PCOS patients (**Figure 6G**). Using flow cytometry to detect cell proliferation also verified the above results (**Figures 6H, I**).

## Discussion

PCOS is generally identified as a metabolic disorder. Abnormal metabolic products, including oxidative stress products caused by breakdown and destruction of fat cells, induce the accumulation of inflammatory cytokines (4). Subsequently, these inflammatory cytokines may accumulate and promote chronic low-grade inflammation. This abnormal accumulation of inflammation seems to impair ovarian function and may aggravate PCOS development.

Early studies have further confirmed that in PCOS patients, peripheral blood circulating CRP and inflammatory cytokines such as IL-6 are increased (31). There have been a significant number of studies on estimating IL-6 levels in association with PCOS, no matter in a vast number of murine models or human subjects (32). This chronic low-grade inflammation promotes the onset of PCOS disease (31). According to our clinical dates collected from PCOS patients and controls, we found an increase in white blood cells in the serum. These immune cells produce large amounts of inflammatory cytokines and result in systemically chronic inflammation (33). Adams et al. found elevated levels of inflammatory cytokines IL-6 and TNF in follicular fluid (34). Our results also revealed that the mRNA level of IL-6 were increased in GCs of PCOS patients. Thus, we conjecture that inflammatory cytokines enter the ovary from blood circulation, and accumulate in follicular fluid from ovarian microcirculation (34).

The latest majority of results demonstrate that levels of typical pro-inflammatory cytokines (including IL-1β and IL-18) in the serum, ovarian and follicular fluid of PCOS were increased significantly (6, 15). Our results also showed that levels of IL-1β and IL-18 in follicular fluid of PCOS patients were increased. However, some research seems to hold the opposite opinion (35). To investigate whether inflammatory cytokines in follicles are predictive factors for PCOS diagnosis, we demonstrated the regression between levels of IL-1β and IL-18 and PCOS diagnosis with ROC curves. We found that the expression of related genes was indeed up-regulated. This is a possible cause of GCs inflammatory initiation in PCOS patients, and may even be the root of PCOS disease progression, but its mechanism remains to be further explored.

PCOS patients are more prone to intestinal flora imbalance and microbiota abnormalities caused by enteral malnutrition, which can producing metabolic endotoxin (LPS) that invades the intestinal wall and aggravates systemic inflammation (36). Therefore, we used LPS as a classic inducer to activate NF-κB pathway to simulate the similar inflammatory status of PCOS patients (19). Thus, we stimulated KGN cells with LPS to promote the expression of inflammasome components (signal 1) and ATP to activate the further assembly of these components (signal 2) (21). As stimulated by LPS, TLR4 was profoundly enhanced in both GCs and KGN cells. Ultimately, we pleasantly found that NF-κB pathway was activated and NLRP3 inflammasome assembled in GCs. Therefore, exogenous LPS may also enter follicular fluid from periphery to further induce inflammatory activation.

A suitable microenvironment is required for oocyte growth and maturation, and the signal transduction between somatic cells and oocytes in follicles constructs a dynamic homeostasis internal environment (37). The microenvironments stability would be controlled by internal and external environments simultaneously (38). To explore whether altered follicular microenvironment provides inflammatory stress to GCs, we incubated KGN cells with follicular fluid in PCOS patients and detected intracellular inflammatory indicators. We found that with treating follicular fluid in PCOS patients, KGN cells showed consistent phenotypes with GCs in PCOS patients. By treating LPS and ATP, NF-κB inflammatory pathways were activated, and NLRP3 inflammasomes were formed in KGN cells. It suggested that internal microenvironment of follicles undergoes adaptive alteration, which induces innate inflammatory responses in GCs.

The physiological conditions of energy metabolism in GCs play an integral role in maintaining oocyte growth (39, 40). Physiologically, pro-inflammatory cytokines produce during follicular development and participate in inducting ovulation, while continuous chronic inflammation can impair follicular growth and affect subsequent reproductive potential (11, 34). Under stimulation of several inflammatory cytokines, GCs make specific physiological responses and adapt to the damage of external factors by altering follicle microenvironment. Excessive and sustained inflammatory stress impacts the function of GCs and inevitably disturbs the quality of oocytes, resulting in subsequent infertility (41). Studies have shown that NF-κB can inhibit the telomerase activity of GCs (42), thereby shortening the lifespan of GCs and preventing oocytes from sufficient nutrients. By producing NLRP3 inflammasomes, this inflammatory response promotes GC pyroptosis and ovarian fibrosis, and disrupts follicular formation (43). While the inflammatory factor IL-1 can inhibit FSHR and LHR, thereby inhibiting follicular maturation and inhibiting ovulation (15). As our study results revealed, inflammation harmed the proliferation of GCs.

**Figure 7:**
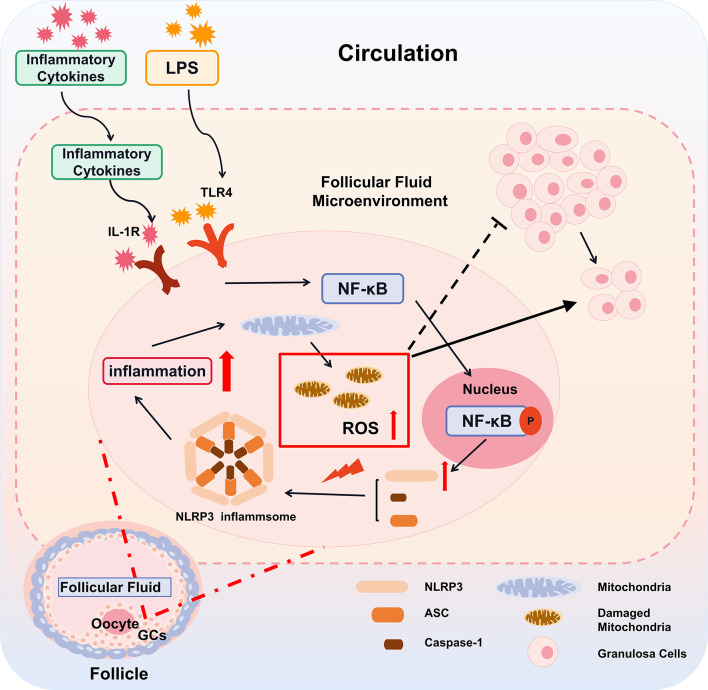
A proposed model for inflammatory cascade in ovarian granulosa cells with PCOS. The inflammatory cytokines derived from the peripheral circulation enter into the follicles through the ovarian circulation system. Subsequently, by the IL-1R and TLR4 on the GCs, the inflammatory cytokines in follicular fluid alternate follicular microenvironment, resulting in the activation of nuclear factors NF-κB and its transfer into the nucleus. Activated NF-κB promotes the gene expression of key components of the NLRP3 inflammasome. Under the stress of mitochondrial ROS, these key components including NLRP3, ASC, caspase-1 assemble, promote the cleavage of IL-1β and amplify the inflammatory cascade. The inflammatory cascade further damage mitochondria, which aggravates the generation of mitoROS and forms a vicious circle. Thereby, the alternation of the follicular microenvironment affects the function of GCs and leads to slowing down of cell proliferation, ultimately affecting the growth and development of oocytes.

However, inflammatory and metabolic diseases are associated with perturbed mitoROS production (44). In this study, we found that with LPS treatment, the mitochondrial morphology of cells was changed significantly, which may be the direct cause of inflammation-induced mitochondrial damage to destroy energy generation of GCs (45). As our results show, the intracellular ROS content also increases significantly after inflammation is induced. Excessive abnormal ROS production would cause redox imbalance in the body, causing mitochondrial structure and function damage (30, 46). Mitochondrial dysfunction may affect follicle growth and development, leading to early follicle atresia (47–49). All the above factors may cause a follicular development disorder in PCOS patients and serve as a key reason for anovulation and sparse ovulation (**Figure 7**).

Because PCOS pathogenesis is unclear and etiology is complicated, it is a technical problem that needs to be overcome urgently. Some scholars believed that inflammation-related signaling pathways are not the main contributors to PCOS (50, 51). Most studies suggested that inflammatory conditions in PCOS are caused by associated obesity or insulin resistance rather than an independent feature of the syndrome (50). Some scholars have pointed out the expression of IL-1β gene in PCOS and non-PCOS was non-significantly different, but overweight PCOS patients had higher levels of IL-1β in serum (35). So whether IL-1β is the cause of PCOS disease remains controversial. According to our study, chronic low-grade inflammation plays an indelible role in its occurrence and development. If this chronic inflammation can be attenuated, it will likely have important significance for improving fertility of PCOS patients. Yang et al., in their work silenced UCA1 and inhibited most pathological progression in PCOS, such as preventing pro-inflammation production and promoting GC proliferation (52). So the target for inflammatory is crucial in PCOS therapy. At present, we have found that alternating follicular microenvironment activated inflammatory pathway, influenced the ability of GCs to proliferation, exacerbates oocyte maturation arrest. Therefore, improving follicular microenvironment can provide a novel direction for treating PCOS patients and remains to be further studied.

## Data Availability Statement

The original contributions presented in the study are included in the article/**Supplementary Material**. Further inquiries can be directed to the corresponding authors.

## Ethics Statement

The studies involving human participants were reviewed and approved by Institutional Review Committee of Guangdong Women and Children Health Hospital of Guangzhou Medical University. The patients/participants provided their written informed consent to participate in this study.

## Author Contributions

YL and HL designed the study. YL and HF wrote the manuscript. YL, ZL, and HL performed the experiments, analyzed, and interpreted the data with the help of other authors. DF revised the article critically for important intellectual content. DF and XW supervised the work. All authors contributed to the article and approved the submitted version.

## Funding

This work was supported in part by the Guangdong Science and Technology Plan Project (grant number: 2014A020212229), the Guangdong Natural Science Foundation (grant numbers: 10151063201000036; S2011010002526), the Guangdong Natural Science Foundation (grant number: 2016A030313760), the Guangzhou Science and Technology Plan Project (grant number: 201804010003), the NSFC (31771531), the Guangzhou Municipal “Ling Nan Ying Jie” Project (2018), and the Chuang Xin Qiang Xiao project of Guangzhou Medical University (2019KCXTD015).

## Conflict of Interest

The authors declare that the research was conducted in the absence of any commercial or financial relationships that could be construed as a potential conflict of interest.

## Supplementary Material

Supplementary Figure 1 The mRNA lever of IL-6 were increased in ovarian granulosa cells of PCOS patients, and up-regulated in KGN cells with LPS stimulation. **(A)** IL-6 mRNA levels between PCOS patients and controls in GCs were measured by RT-qPCR assays (P = 0.0071). **(B)** With LPS (200 ng/mL) stimulation in KGN cells, IL-6 mRNA levels were detected by RT-qPCR assays (4 h: P=0.0073, 6 h: P = 0.0374). *P < 0.05, **P < 0.01. *P < 0.05 was considered statistically significant.
