# Supplementary figures and images for: The Release of Peripheral Immune Inflammatory Cytokines Promote an Inflammatory Cascade in PCOS Patients via Altering the Follicular Microenvironment
Source: Front Immunol. 2021 May 17;12:685724. doi: 10.3389/fimmu.2021.685724 (PMC8165443; doi:10.3389/fimmu.2021.685724)

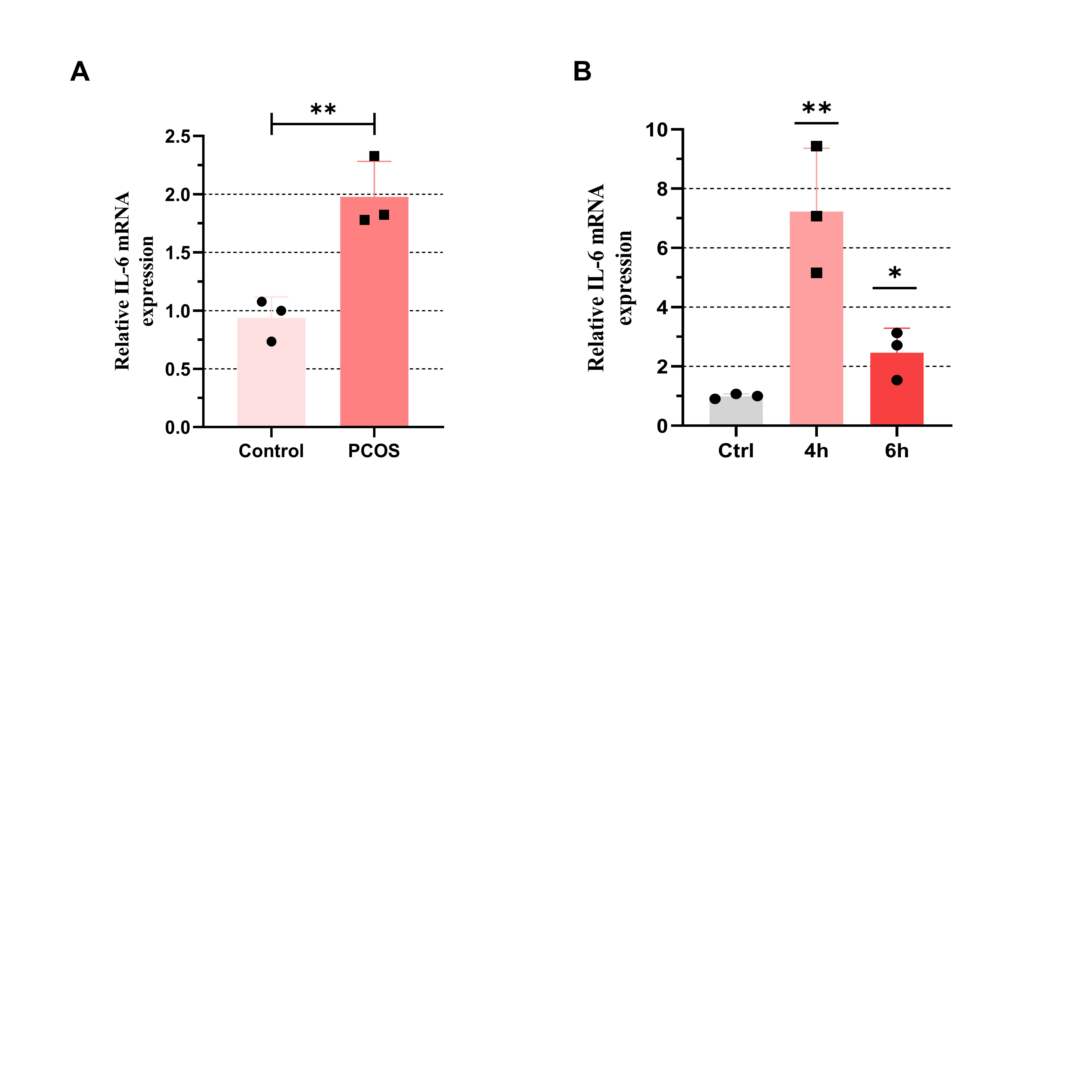

Supplement: Supplementary Figure 1 — The mRNA lever of IL-6 were increased in ovarian granulosa cells of PCOS patients, and up-regulated in KGN cells with LPS stimulation. (A) IL-6 mRNA levels between PCOS patients and controls in GCs were measured by RT-qPCR assays (P = 0.0071). (B) With LPS (200 ng/mL) stimulation in KGN cells, IL-6 mRNA levels were detected by RT-qPCR assays (4 h: P=0.0073, 6 h: P = 0.0374). *P < 0.05, **P < 0.01. *P < 0.05 was considered statistically significant. [file Image_1.tif]
